# Dietary characterization of the endangered salt marsh harvest mouse and sympatric rodents using DNA metabarcoding

**DOI:** 10.1002/ece3.9121

**Published:** 2022-07-17

**Authors:** Cody M. Aylward, Mark J. Statham, Laureen Barthman‐Thompson, Douglas A. Kelt, Benjamin N. Sacks

**Affiliations:** ^1^ Department of Wildlife, Fish, and Conservation University of California, Davis Davis California USA; ^2^ Mammalian Ecology and Conservation Unit, Veterinary Genetics Laboratory, School of Veterinary Medicine University of California, Davis Davis California USA; ^3^ California Department of Fish and Wildlife Stockton California USA; ^4^ Department of Population Health and Reproduction, School of Veterinary Medicine University of California, Davis Davis California USA

**Keywords:** endangered species, metabarcoding, noninvasive diet analysis, *Reithrodontomys raviventris*, seasonal diet analysis

## Abstract

The salt marsh harvest mouse (*Reithrodontomys raviventris*; RERA) is an endangered species endemic to the coastal wetlands of the San Francisco Estuary, California. RERA are specialized to saline coastal wetlands, and their historical range has been severely impacted by landscape conversion and the introduction of non‐native plant and rodent species. A better understanding of their diet is needed to assess habitat quality, particularly in relation to potential competitors. We investigated three questions using DNA metabarcoding with ITS2 and trnL markers: (1) Do RERA specialize on the native plant, pickleweed (*Salicornia pacifica*), (2) Do RERA consume non‐native plants, and (3) What is the dietary niche breadth and overlap with three sympatric native and non‐native rodents? RERA diet was dominated by two plants, native *Salicornia* and non‐native salt bush (*Atriplex* spp*.*), but included 48 plant genera. RERA diet breadth was narrowest in fall, when they consumed the highest frequencies of *Salicornia* and *Atriplex*, and broadest in spring, when the frequencies of these two plants were lowest. Diet breadth was slightly lower for RERA than for co‐occurring species in pairwise comparisons. All four species consumed similarly high frequencies of wetland plants, but RERA consumed fewer grasses and upland plants, suggesting that it may be less suited to fragmented habitat than sympatric rodents. Diet overlap was lowest between RERA and the native California vole (*Microtis californicus*). In contrast, RERA diet overlapped substantially with the native western harvest mouse (*R. megalotis*) and non‐native house mouse (*Mus musculus*), suggesting potential for competition if these species become sufficiently abundant.

## Graphical abstract

We used DNA metabarcoding to characterize the diet of the endangered salt marsh harvest mouse (*Reithrodontomys raviventris*) and three sympatric rodents. We found that salt marsh harvest mice had a broad diet but appeared to prefer pickleweed (*Salicornia pacifica*) and fat‐hen (*Atriplex prostrata*). Salt marsh harvest mouse diet strongly overlapped sympatric generalist rodents, but was more restricted, suggesting that dietary competition and habitat fragmentation are potential threats to salt marsh harvest mice.

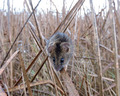

## 1 INTRODUCTION

**FIGURE 1:**
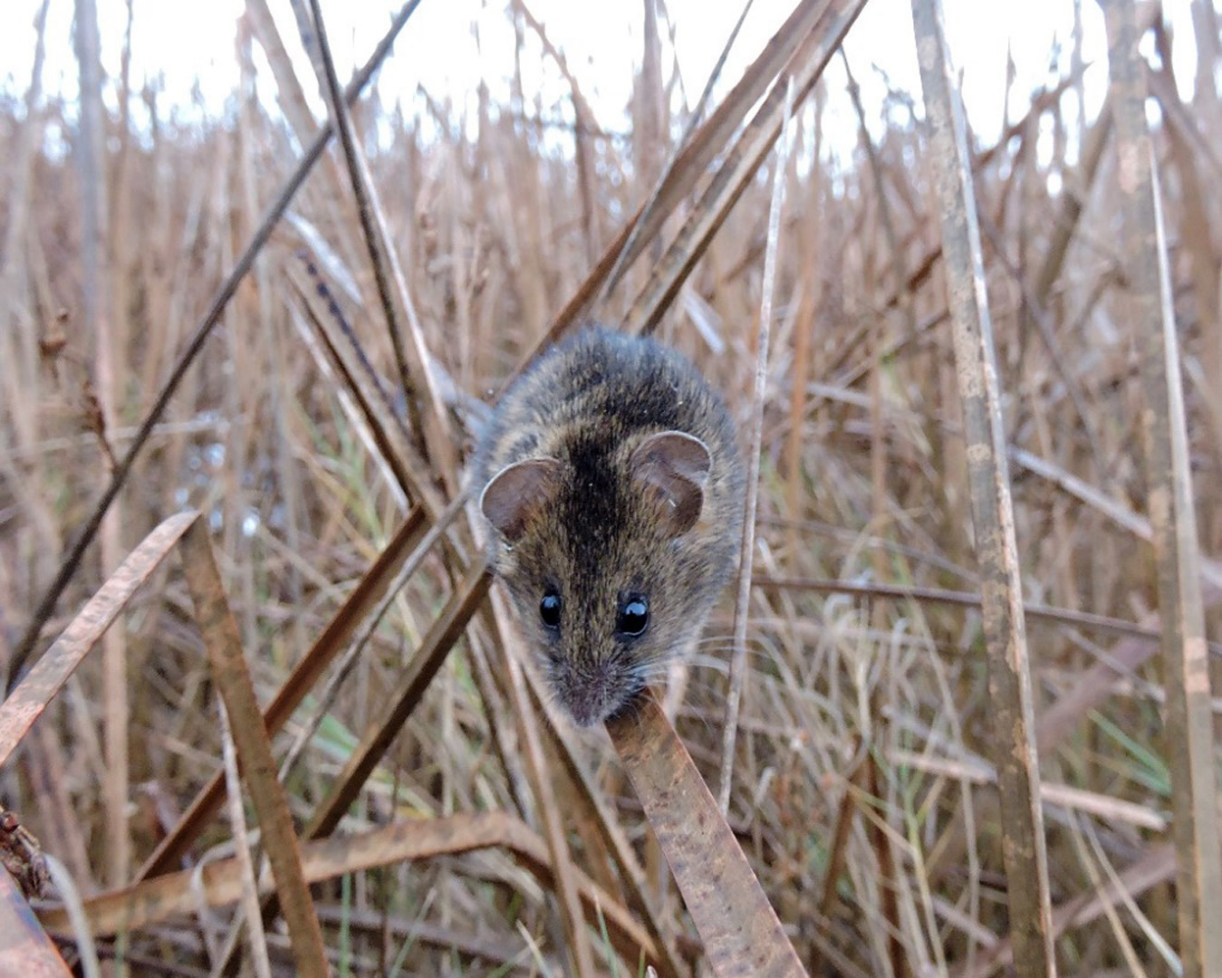
Salt marsh harvest mouse (*Reithrodontomys raviventris)* in salt marsh habitat in Suisun marsh, California, USA

The salt marsh harvest mouse (*Reithrodontomys raviventris*; RERA; Figure 1) is a habitat specialist occurring solely in the salt marshes of the San Francisco Estuary (SFE), California, USA. They are the only known mammal species restricted to coastal marshes (Greenberg et al., 2006). Despite being listed as an Endangered Species since the inception of the US Endangered Species Act (United States Fish and Wildlife Service [USFWS], 1970), the ecology of RERA remains poorly known. Most prior research effort has emphasized habitat associations (Smith et al., 2018a). Other aspects of RERA ecology, including diet, predation, disease ecology, and interspecific interactions, remain poorly understood (Smith et al., 2018b). Historically, RERA has been considered a specialist of *Salicornia* marsh habitat (Fisler, 1965; Shellhammer et al., 1982; USFWS, 1984). Recent evidence, however, suggests that RERA may be less specialized to *Salicornia* habitat than believed, particularly in brackish marshes with lower salinity and greater plant diversity (Smith et al., 2020; Smith & Kelt, 2019; Sustaita et al., 2011). The SFE has been altered by over a century of anthropogenic impacts, including the loss of >90% of historical tidal marsh habitat (Hobbs et al., 2006; Williams & Faber, 2001). Reflecting these threats, RERA is listed as endangered by the state of California (California Department of Fish and Wildlife [CDFW], 1971), the federal government (USFWS, 1970), and the International Union for Conservation of Nature (IUCN, 2021).

Three other rodent species are commonly detected in SFE marshes, the western harvest mouse (*R. megalotis*; REME), California vole (*Microtus californicus*; MICA), and house mouse (*Mus musculus*; MUMU). REME is native to this region, ranges throughout the western United States, and is considered a habitat generalist (Webster & Jones, 1982). REME are less salt tolerant than RERA and are thought to occur primarily in uplands and marsh‐upland edge in the SFE (Fisler, 1965). MICA also is native and is considered a grassland specialist (Batzli & Pitelka, 1971). MICA are notably larger than both harvest mouse species, and likely are behaviorally dominant to them, although RERA may be better adapted to high salinity conditions (Blaustein, 1980; Geissel et al., 1988). Previous work has indicated both negative (Geissel et al., 1988) and positive (Sustaita et al., 2011) associations between RERA and MICA habitat use, but their diet interactions remain unknown. Finally, non‐native MUMU commonly co‐occur with RERA throughout the SFE (e.g., Bias & Morrison, 2006; Marcot et al., 2020). MUMU are highly fecund (Bronson, 1979; Pye, 1993), opportunistic, and tolerant of a wide range of ecological conditions (Berry, 1981). MUMU and RERA are compatible in captivity (Catlett & Shellhammer, 1962), and may (Bias & Morrison, 2006) or may not (Sustaita et al., 2011) partition habitat, but the recapture probabilities for MUMU were positively influenced by RERA densities at Suisun Marsh (Smith et al., 2020).

Non‐native plants and animals are abundant in remnant SFE marshes (Grewell et al., 2014; Smith & Kelt, 2019; USFWS, 2010). Non‐native species can affect multiple trophic levels of a community, as they may represent novel predators, novel competitors, or novel food resources to different community members (Lepczyk & Rubinoff, 2017). Ecological specialists may be particularly sensitive to non‐native species, as they may be less likely than generalists to utilize novel resources (Abernethy et al., 2016; Marvier et al., 2004). Indeed, the ecological impacts of non‐native species are considered the leading cause of extinction for endemic mammals (Pimm et al., 1995). Given the challenges that ecological specialists must overcome to persist in a highly altered ecosystem, understanding the effects of non‐native food resources and non‐native intraguild species are critical ecological underpinnings for RERA conservation.

Despite the importance of strong ecological baselines to conservation and management of endangered species, the dietary habits of RERA remain poorly understood. RERA diet has been inferred from habitat use (USFWS, 2010), characterized coarsely by stomach content analysis (Fisler, 1965), and measured with cafeteria trials (Smith & Kelt, 2019). Rather than providing consensus, these studies have led to divergent views of RERA diet. Habitat associations and stomach content analyses suggest that RERA consume primarily *Salicornia* (Fisler, 1965). Conversely, cafeteria trials suggest that RERA are generalist foragers that may prefer some non‐native plant species over *Salicornia*. Additionally, comparative dietary interactions of RERA and sympatric rodents have never been investigated.

To address these critical knowledge gaps, we applied DNA metabarcoding to fecal samples collected from rodents in the SFE. Metabarcoding often identifies significantly more dietary taxa at finer taxonomic levels than other methods (Kartzinel et al., 2015; Soininen et al., 2009; Valentini et al., 2009). Additionally, the noninvasive nature of dietary metabarcoding makes it particularly appealing for research on threatened and endangered species (e.g., Castle et al., 2020; Iwanowicz et al., 2016). Our objectives were to describe diets of RERA and sympatric rodents and characterize both spatial and temporal dietary variation. We evaluated the hypothesis that RERA has a more specialized diet than sympatric species, and assessed the potential for competition over food resources.

## 2 METHODS

### 2.1 Sample collection

**FIGURE 2:**
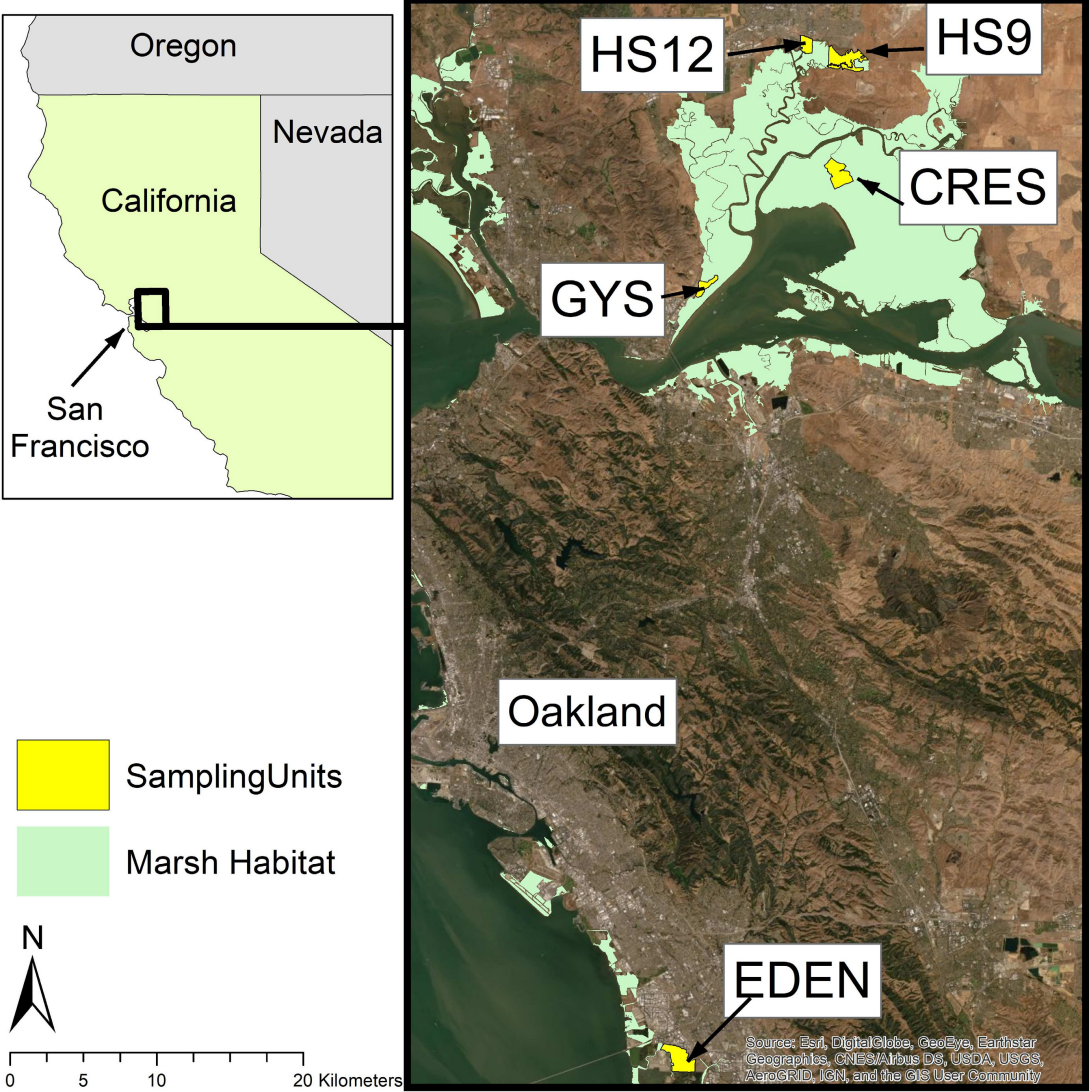
Map of sampling locations in this study. Five sites were live‐trapped for salt marsh harvest mice (*Reithrodontomys raviventris*) and other small mammals and fecal pellets were collected to characterize diet with DNA metabarcoding. Sites included Goodyear Slough (GYS) and crescent unit (CRES) of the Grizzly Island wildlife area, ponds 1&2 (HS12) and area 9 (HS9) of the Hill Slough wildlife area, and EDEN landing ecological reserve (EDEN). GYS was sampled quarterly over two years to provide seasonal dietary data. All other sites were trapped opportunistically on one occasion each, either in late spring (CRES and EDEN) or summer (HS12 and HS9)

We collected feces from animals captured during regular live‐trapping surveys (see Smith et al., 2020 for details of survey design and associated protocols). Preliminary trials showed that feces collected directly from live‐trapped animals were more likely to be composed entirely of diet items from the trapping bait, so we endeavored to collect feces from the bedding in traps to characterize diet before consumption of bait. Samples were collected at five sites in coordination with the California Department of Fish and Wildlife during regular RERA monitoring (Figure 2). One of these sites—the Goodyear Slough Unit (GYS) of the Grizzly Island Wildlife Area—was trapped quarterly over 2 years (Summer 2018–Spring 2020, inclusive), allowing us to partition diet into four seasonal data sets; all other sites were trapped once either in summer or late spring, resulting in a total of eight sampling units (Table 1). We also surveyed vegetation plots to characterize availability of potential diet items at sampling sites/seasons. Within 2 weeks of each live‐trapping effort, we recorded the presence of all plant genera in 3‐m × 3‐m quadrats centered at the location of each trap. All methods involving live animals followed guidelines of the American Society of Mammalogists (Sikes & Animal Care and Use Committee of the American Society of Mammalogists, 2016), were approved by the UC Davis Institutional Animal Care and Use Committee, and conducted under authority of the Cooperative Agreement between California Department of Fish and Wildlife (CDFW) and the United States Fish and Wildlife Service.

**TABLE 1:** Number of individuals of four rodent species captured during each season at each site: Salt marsh harvest mouse (*Reithrodontomys raviventris*; RERA), western harvest mouse (*R. megalotis*; REME), house mouse (*Mus musculus*; MUMU), and California vole (*Microtus californicus*; MICA)

	Sampling unit								
Species	GYS (Su)	GYS (fa)	GYS (Wi)	GYS (Sp)	HS12	HS9	CRES	EDEN	Total
RERA	45^a^, ^b^, ^c^	52^a^, ^b^	48^a^	44^b^	8^c^	14	13	21^b^, ^c^	245
REME	13^a^	7^a^	7^a^	2	0	1	0	0	30
MUMU	8^c^	4^c^	1	1	7^c^	0	1	4^c^	26
MICA	4^b^	0	0	10^b^	0	0	1	5^b^	20

*Note*: Goodyear Slough (GYS) was surveyed during all four seasons (Su = summer, fa = fall, Wi = winter, and Sp = spring), whereas Hill Slough 1&2 (HS12), Hill Slough 9 (HS9), crescent unit (CRES), and EDEN landing (EDEN) were sampled only in summer or late spring. Due to varying sample sizes by site and season, interspecific comparisons of diet were conducted on a pairwise basis between RERA and one other species independently, and only at sites where diet data were available for ≥4 individuals of both species.

^a^ Sites with sufficient sample size to be included in comparisons of RERA and REME diet.

^b^ Site included in RERA / MICA comparisons.

^c^ Site included in RERA / MUMU comparisons.

### 2.2 Laboratory procedures

We extracted DNA from fecal samples using Qiagen Plant Mini Kits (Qiagen, CA, USA). For each captured individual, we extracted DNA from pooled fecal pellets; we targeted >5 pellets from each individual, and final pellet numbers in extractions ranged from 1–13 (mean = 5.7). Library preparation followed the general template of the Illumina 16S metagenomic protocol (Illumina, 2015). Since single markers may only amplify a subset of plant taxa in herbivore diets (Goldberg et al., 2020), we applied two commonly used plant metabarcoding markers. We amplified the second internal transcribed spacer (ITS2), which is a longer fragment (~290–340 bp) of nuclear ribosomal DNA with high taxonomic resolution (China Plant Barcode of Life Group et al., 2011), and the P6 loop of the trnL intron, a shorter fragment (~25–90 bp) of chloroplast DNA, which is less likely to be affected by degradation but has coarser taxonomic resolution (Fahner et al., 2016). We used the R package “PrimerMiner” (Elbrecht & Leese, 2016) to evaluate the compatibility of potential primer pairs with sequences of suspected RERA dietary taxa (based on vegetation surveys and the Suisun Marsh Plant List; CDFW, 2017) downloaded from Genbank. We used the primers UniPlantF (Moorhouse‐Gann et al., 2018) and ITS‐P4 (Cheng et al., 2016) for ITS2, and the primers trnl_g and trnl_h (Taberlet et al., 2007) for trnL. We added sequence overhangs to the 5′ ends of amplicon primers to facilitate annealing to Illumina sequencing adapters (compete primer sequences in Appendix A1, PrimerMiner scores in Appendix A2). We amplified ITS2 using the thermal protocol described in Moorhouse‐Gann et al. (2018) and amplified trnL using the thermal protocol described in Taberlet et al. (2007). Given that biological replication (i.e., samples from unique individuals) yields significantly more variation in diet than technical replication (i.e., multiple PCR replicates per individual), we chose to prioritize our resources for biological replication and therefore conducted a single PCR replicate for each individual (Mata et al., 2019).

We included 20 positive controls and 12 negative controls per sequencing lane (Appendix A3). Positive controls were composed of DNA extracted from plants collected from our field sites. Each set of 20 positive controls included 10 single‐species controls to assess sensitivity and to help estimate misassignment error based on the proportion of nontarget reads within single‐species controls. We also included 10 two‐species controls, which had equal concentrations of DNA from two plant taxa and helped to understand potential amplification biases. We used deionized water for negative controls. We sequenced libraries using MiSeq 300 PE for ITS2 and 75 PE for trnL. Sequencing and sample demultiplexing were conducted by the UC Davis Genome Center.

### 2.3 Bioinformatic processing

We trimmed and quality‐filtered sequences using cutadapt (Martin, 2011). We identified Amplicon Sequence Variants (ASVs) using DADA2 (Callahan et al., 2016). To identify the taxonomy of ASVs, we created a custom database of ITS2 and trnL sequences of all plant genera known to occur in the SFE using the *batch_download* feature of PrimerMiner, which obtains sequences from both NCBI (Benson et al., 2013) and BOLD (Ratnasingham & Hebert, 2007) databases, and we manually re‐formatted the reference sequences for use in DADA2. We used the *assignTaxonomy* feature of DADA2 to assign ASVs against the custom database, and used BLAST (Zhang et al., 2000) to corroborate assignments. We assigned ASVs at the genus level, except for some trnL sequences that could not be assigned to a single genus and were therefore assigned to the lowest possible suprageneric level (e.g., family or multiple genera).

We conducted sequence processing and assignment independently for each MiSeq lane. After taxonomic assignment, we retained only ASVs that comprised >0.01% of the total sequence reads in a lane. We then used positive and negative controls to inform filtering parameters to account for misassigned ASVs (O'Rourke et al., 2020). Based on the negative and positive controls, we discarded any sample with <5000 (trnL) or <3000 (ITS2) sequencing reads, or with <20% of reads successfully matching plant taxa; and within samples, we discarded any taxa comprising <0.5% (trnL) or <1.0% (ITS2) of reads. After applying those filters, we removed any taxa that likely originated from a source other than wild RERA diet (*Avena*, *Helianthus*, *Juglans*, *Panicum*, and *Phalaris* from trapping bait, and *Gossypium* from trap bedding).

### 2.4 Salt marsh harvest mouse diet

We recorded the presence/absence of diet taxa within individual diets. We calculated the frequency of occurrence (FO = the proportion of individuals that consumed a given diet item) of diet items within RERA samples pooled across all sampling locations and seasons. We then categorized diet items as native versus non‐native, by life form (grass, shrub, forb, vine), and by habitat (e.g., whether they were typical of wetlands or of uplands; determined from Jepson eFlora [Jepson Flora Project, 2021]), and we estimated FO for each category. We chose to use the presence/absence‐based data (i.e., FO) due to the complexity of estimating biomass from relative read abundance (e.g., Deagle et al., 2019) with two different markers that detected different suites of species (see Appendix B). Our sample sizes were sufficient to produce a strong correlation between FO and RRA (Appendix A4), so use of FO was unlikely to affect downstream analyses. We estimated plant availability at the site level by calculating FO of plant genera among all quadrats at a site.

We evaluated seasonal variation in RERA diet at Goodyear Slough. For this analysis, we pooled data by season across the 2 years, and estimated FO of diet items within each season. We tested for significant seasonal differences using the *anosim* function in the “vegan” R package (Oksanen et al., 2020). We compared diet to plant availability with Manly's Selection Index (*W*_*i*_; Manly et al., 2002) using the R package “adehabitatHS” (Calange, 2011). We considered diet items to be “selected” when *W*_*i*_ ± 95% confidence intervals >1, and “avoided” when *W*_*i*_ ± 95% CI <1. Since vegetation availability data were collected at the genus level, we excluded any diet items identified at a coarser taxonomic level from selection analyses. We quantified dietary niche breadth as the effective number of species (^1^D; Hill, 1973; Chao et al., 2014) derived from Shannon's Diversity Index (Shannon & Weaver, 1949) for unequal sample sizes and presence/absence data (Chao et al., 2014). We used the R package “iNEXT” (Chao et al., 2014) to estimate ^1^D and 95% CIs using 500 bootstrap replicates. We considered seasonal differences in dietary niche breadth significant if 95% CIs were non‐overlapping. Additionally, we estimated dietary niche overlap between pairs of seasons using Jaccard's Similarity Index (*J*_*s*_), calculated in “vegan.” To visualize dietary niches in ordination space, we conducted non‐metric multidimensional scaling (nMDS) based on Jaccard Distances (*J*_*D*_) using the metaMDS function in “vegan,” and calculated 95% confidence ellipses for each season. We conducted nMDS over a range of dimensions (*k*) and selected the minimum number of dimensions (*k* = 3) in which stress of the ordination was <0.10.

We evaluated spatial variation in RERA diet from five sites sampled in late spring and summer (henceforth, “summer” sampling units): Goodyear Slough (GYS; summer) and Crescent Unit (CRES; late spring) of the Grizzly Island Wildlife Area, Ponds 1&2 (HS12; summer) and Area 9 (HS9; summer) of the Hill Slough Wildlife Area, and Eden Landing Ecological Reserve (EDEN; late spring). We estimated the mean and variance of FO for each diet item, and the mean and variance of plant availability, across the five sampling units. We calculated *W*_*i*_ of diet items within each sampling unit and combined across all sampling units.

### 2.5 Diet of salt marsh harvest mice and sympatric rodents

We calculated FO for all diet items and for all rodent species pooled across all sampling units. Further comparative analyses (dietary niche breadth and overlap) were limited to pairwise comparisons with RERA and used only those sampling units where we had dietary information for >4 individuals of both species. To ensure that large sample sizes at one site (i.e., Goodyear Slough) did not bias interpretations of dietary niche breadth and overlap, we calculated FO of diet items at individual sites and then averaged these site‐level FOs, thereby giving equal weight to the population‐level diet information at each site. We compared RERA diets to REME (three sampling units), MICA (three), and MUMU (four). We calculated ^1^D and *J*_*s*_ for comparison across each species pair and considered dietary niche breadth significantly difference if 95% CIs of ^1^D were non‐overlapping. Additionally, we visualized dietary niches of species in ordination space with nMDS based on *J*_*D*_, and we used 95% confidence ellipses to qualitatively assess diet overlap.

## 3 RESULTS

### 3.1 Field sampling

We collected fecal samples from 327 unique individuals from the eight sampling units. Six samples were discarded during bioinformatic filtering (see Appendix B), leaving 321 samples for subsequent analyses. Sample sizes were significantly larger for RERA (*n* = 245) than for REME (*n* = 30), MUMU (*n* = 26), or MICA (*n* = 20). Sample sizes also were heavily weighted toward Goodyear Slough (*n* = 246) due to quarterly sampling over 2 years. All plants used in controls were reliably detected with the exception of *Schoenoplectus*, which is considered a likely food item for RERA but was absent from our results.

### 3.2 Salt marsh harvest mouse diet

**FIGURE 3:**
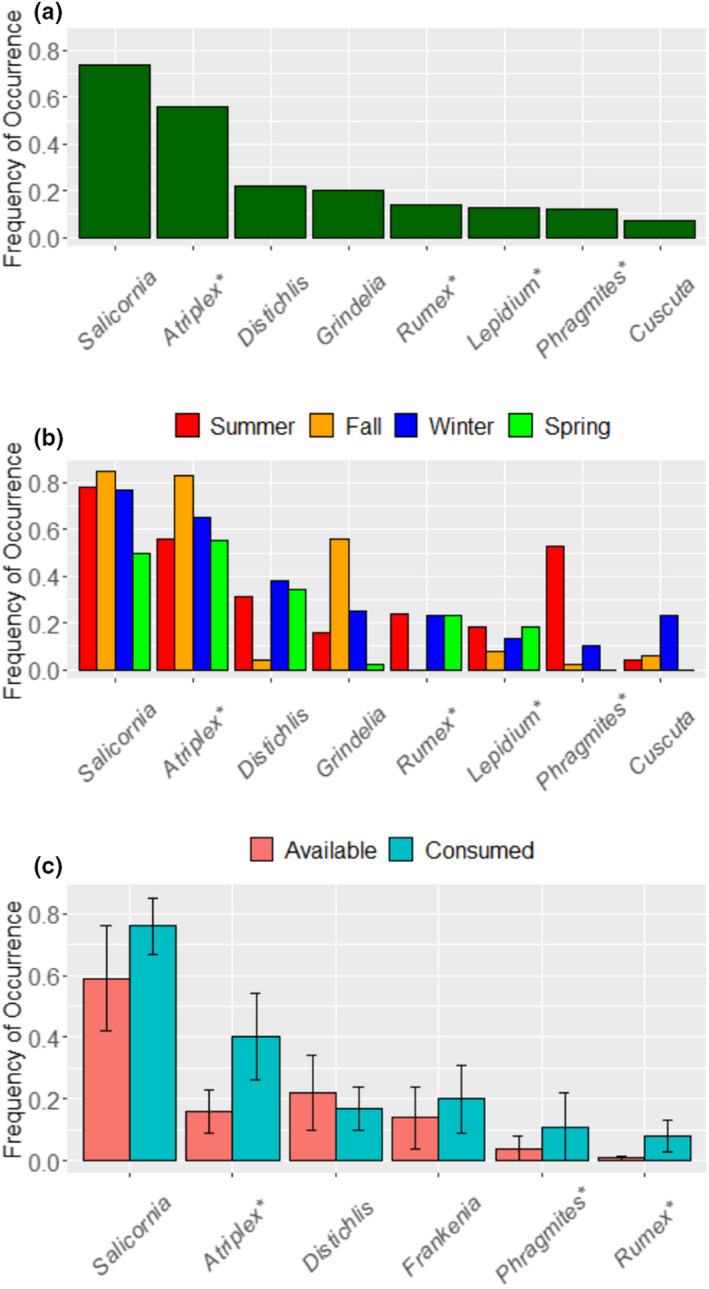
Frequency of occurrence (FO) of plant genera consumed by salt marsh harvest mice (*Reithrodontomys raviventris*). (a) FO of the eight most frequently consumed plants pooled across all sites and seasons (*n* = 245 individuals). Over 40 additional genera were consumed at lower frequencies. (b) Seasonal FO of the top eight plants consumed at Goodyear Slough. (c) Mean and SE of FO in diets compared to FO in vegetation quadrats sampled at five summer sampling units. In all panels, non‐native plants are denoted with an asterisk (*)

We documented 53 taxa, including 48 genera and 5 higher order identifications in the diet of RERA (Appendix A5). When data were pooled across all eight sampling units, seven plant genera presented a FO > 10% (Figure 3a). *Salicornia* and *Atriplex* stood out from the rest of the dataset (FO > 0.50), and *Distichlis*, *Grindelia*, *Rumex*, *Lepidium*, and *Phragmites* had moderate FOs (>0.10). RERA diet was dominated by wetland forbs/subshrubs (Appendix A6), and both native and non‐native items were prevalent.

**FIGURE 4:**
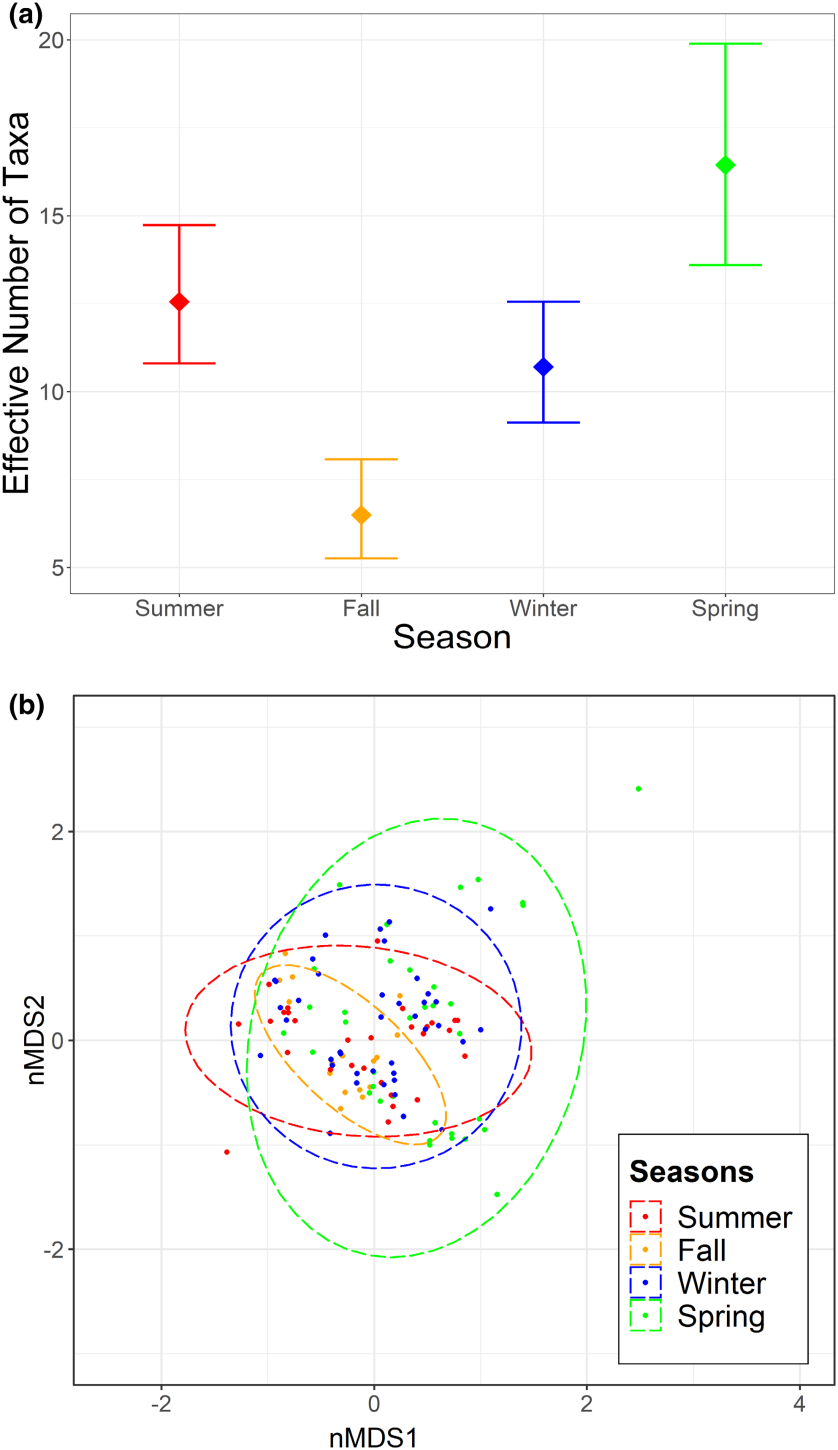
(a) Dietary niche breadth (effective number of taxa; ^1^D; Hill, 1973) of salt marsh harvest mouse (*Reithrodontomys raviventris*) diet in four seasons over two years at Goodyear Slough. (b) Non‐metric multidimensional scaling ordination of seasonal salt marsh harvest mouse (*Reithrodontomys raviventris*) diet at Goodyear Slough. Dots represent individual animals, and dashed lines represent 95% confidence ellipses

Diets at Goodyear Slough varied seasonally (ANOSIM; *R* = .173, *p* = .001). *Salicornia* and *Atriplex* were consumed at high frequencies year‐round, whereas several taxa were consumed either at moderate frequency year‐round (*Lepidium*) or at high frequency but seasonally (*Distichlis*, *Grindelia*, *Rumex*, *Phragmites*, and *Cuscuta*) (Figure 3b; Appendix A7). Wetland forbs/subshrubs were eaten frequently in all seasons, whereas upland plants (grasses and forb/subshrubs) were consumed primarily in spring (Appendix A6). RERA selected five diet items in at least one season (Table 2A; Figure 3c). *Salicornia* and *Atriplex* were each selected in three seasons, and were never avoided. Three genera were selected in summer (*Phragmites*, *Rumex*, and *Salicornia*), fall (*Atriplex*, *Grindelia*, and *Salicornia*), or winter (*Atriplex*, *Rumex*, and *Salicornia*), and only one in spring (*Atriplex*). RERA avoided *Juncus* in all seasons, and *Distichlis* (summer and fall) and *Phragmites* (fall and spring) in two seasons. Combining data across all seasons, RERA selected *Atriplex*, *Grindelia*, *Rumex*, and *Salicornia*, and avoided *Distichlis* and *Juncus*. Dietary niche breadth (^1^D) was significantly lower in fall than all other seasons (Figure 4a). Fall and spring exhibited the lowest similarity (*J*_*s*_) with respect to other seasons (Table 3). Seasonal diets overlapped in ordination (nMDS) space, although confidence ellipses varied in breadth in accordance with estimates of seasonal dietary niche breadth (Figure 4b).

**TABLE 2:** Manly's selection index (*W*_*i*_) for plant genera in salt marsh harvest mouse (*Reithrodontomys raviventris*) diets in (a) four seasons at Goodyear Slough, and (b) summer at 5 locations/sites: Goodyear Slough (GYS), Hill Slough 1&2 (HS12), Hill Slough 9 (HS9), crescent unit (CRES), and EDEN landing (EDEN)

A					
Seasonal	Manly's selection index (*Wi*)				
Genus	Summer	Fall	Winter	Spring	All seasons
*Atriplex*	1.45 (0.91, 1.99)	2.43 (1.90, 2.97)*	2.36 (1.61, 3.10)*	3.36 (1.90, 4.82)*	2.30 (1.90, 2.70)*
*Distichlis*	0.58 (0.25, 0.90)†	0.06 (−0.07, 0.20)†	0.73 (0.32, 1.12)	0.64 (0.25, 1.03)	0.49 (0.31, 0.67)†
*Grindelia*	0.88 (−0.10, 1.86)	6.57 (4.34, 8.80)*	1.56 (0.37, 2.75)	0.13 (−0.28, 0.53)†	1.84 (1.06, 2.61)*
*Juncus*	0.00 (0.00, 0.00)†	0.00 (0.00, 0.00)†	0.07 (−0.15, 0.29)†	0.07 (−0.14, 0.30)†	0.04 (−0.05, 0.12)†
*Phragmites*	3.40 (1.79, 5.00)*	0.17 (−0.34, 0.68)†	0.68 (−0.21, 1.56)	0.00 (0.00, 0.00)†	1.12 (0.52, 1.72)
*Rumex*	8.95 (2.52, 15.38)*	0.00 (0.00, 0.00)†	6.85 (1.33, 12.38)*	7.70 (0.80, 14.60)	6.76 (3.44, 10.07)*
*Salicornia*	1.37 (1.08, 1.66)*	1.32 (1.04, 1.60)*	1.29 (1.04, 1.74)*	0.82 (0.46, 1.17)	1.27 (1.10, 1.45)*

B						
Spatial	Manly's selection index (*Wi*)					
Genus	GYS	HS12	HS9	CRES	EDEN	All sites
*Atriplex*	1.45 (0.91, 1.99)	2.96 (1.62, 4.31)*	4.45 (−8.10, 16.99)	3.53 (−0.06, 7.12)	1.26 (−2.17, 4.69)	2.14 (1.35, 2.93)*
*Distichlis*	0.58 (0.25, 0.90)†	0.82 (−0.75, 2.38)	0.72 (−0.11, 1.56)	‐‐	‐‐	0.72 (0.35, 1.10)
*Frankenia*	‐‐	1.83 (0.29, 3.38	4.45 (−8.10, 16.99)	‐‐	1.13 (0.36, 1.90)	2.79 (1.30, 4.29)*
*Phragmites*	3.40 (1.79, 5.00)*	‐‐	‐‐	‐‐	‐‐	3.52 (1.81, 5.23)*
*Rumex*	8.95 (2.52, 15.38)*	0.00 (0.00, 0.00)†	‐‐	12.35 (−9.19, 33.90)	‐‐	8.61 (2.34, 14.87)*
*Salicornia*	1.37 (1.08, 1.66)*	2.56 (0.15, 4.97)	1.73 (0.35, 3.10)	0.79 (0.40, 1.17)	1.33 (0.74, 1.91)	1.79 (1.38, 2.20)*

*Note*: Tables include all diet items with significant selection (*; *W*_*i*_ ± 95% CI > 1) or avoidance (†; *W*_*i*_ ± 95% CI < 1) in at least one season/site or when all sites/seasons were pooled. Dashes (‐‐) indicate a diet item that was absent from the site and therefore does not have a selection coefficient.

**TABLE 3:** Measures of Jaccard similarity (*J*_*s*_; range 0–1) between seasonal diet of salt marsh harvest mice (*Reithrodontomys raviventris*) at Goodyear Slough

Season	Jaccard's similarity (*J*_s_)				
	Summer	Fall	Winter	Spring	Mean
Summer	–	0.396	0.569	0.476	0.480
Fall		–	0.515	0.291	0.401
Winter			–	0.448	0.511
Spring				–	0.405

At all five summer sampling units, RERA frequently consumed *Salicornia* (FO ≥ 0.50; mean FO = 0.76; Figure 3c; Appendix A8). *Atriplex* (mean FO = 0.40) had a FO ≥ 0.50 in two of five sampling units. *Frankenia*, *Lepidium*, and *Phragmites* were the only other taxa with FO ≥ 0.50 at any given sampling unit. Pooling samples across all summer sites, RERA selected *Atriplex*, *Frankenia*, *Phragmites*, *Rumex*, and *Salicornia* (Table 2B).

### 3.3 Comparison of diet to co‐occurring rodents

**FIGURE 5:**
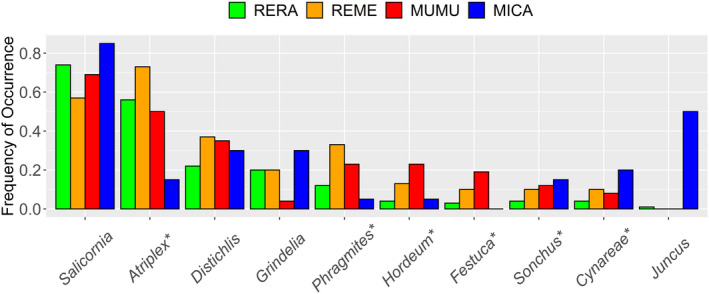
Frequency of occurrence of 10 important diet items in salt marsh harvest mouse (*Reithrodontomys raviventris*; RERA), western harvest mouse (*R. megalotis*; REME), house mouse (*Mus musculus*; MUMU), and California vole (*Microtus californicus*; MICA) diets. For data across all dietary items, see Appendix A9. In all panels, non‐native plants are denoted with an asterisk (*)

The FOs of REME and MUMU were qualitatively similar to those of RERA (Figure 5; Appendix A9). The dominant items in RERA diet (*Salicornia* and *Atriplex*) were also the two most frequently consumed foods by REME (FO = 0.57 and 0.73, respectively) and MUMU (FO = 0.69 and 0.50, respectively). *Salicornia* was the most frequently eaten food by MICA (FO = 0.85), but *Atriplex* was relatively sparse in their diets (FO = 0.15). Notably, grasses (e.g., *Distichlis*, *Phragmites*, *Hordeum*, and *Festuca*) and upland plants (e.g., *Sonchus*, Cynareae) were more prominent in the diets of sympatric rodents than that of RERA. MICA diet was the most distinct, driven by a low frequency of *Atriplex* and high frequency of rushes (*Juncus*).

**FIGURE 6:**
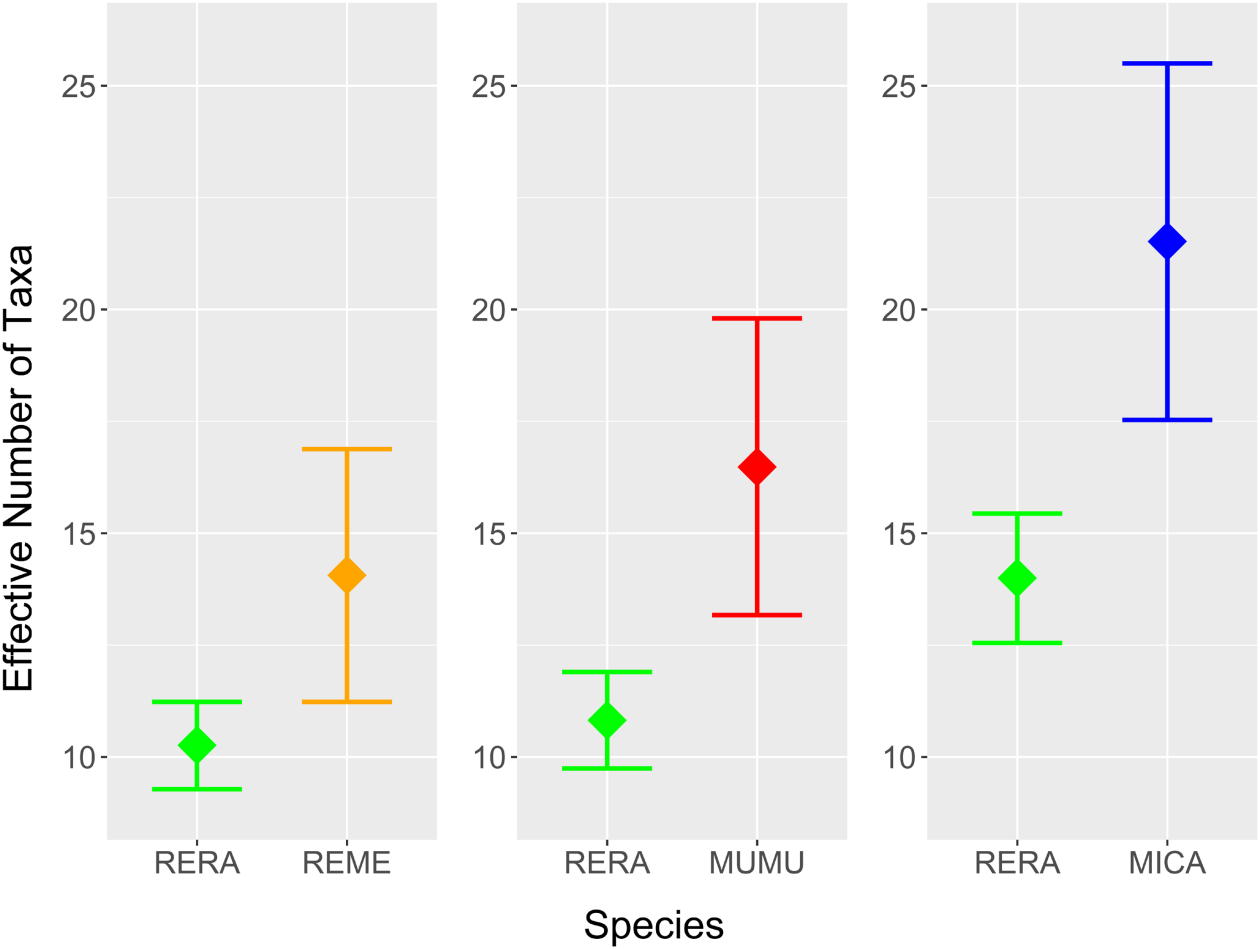
Pairwise comparisons of dietary niche breadth (effective number of taxa; ^1^D; Hill, 1973) of salt marsh harvest mouse (*Reithrodontomys raviventris*; RERA), western harvest mouse (*R. megalotis*; REME), house mouse (*Mus musculus*; MUMU), and California vole (*Microtus californicus*; MICA). Comparisons were conducted pairwise because sample sizes of non‐RERA were inconsistent throughout space and time, therefore only allowing valid comparisons at a different suite of sites/seasons for each species pair. RERA/REME comparisons were conducted at Goodyear Slough (GYS) in summer, fall, and winter; RERA/MUMU comparisons were conducted at GYS (summer and fall), Hill Slough wildlife area ponds 1&2 (summer) and EDEN landing ecological reserve (EDEN; spring); and RERA/MICA comparisons were conducted at GYS (summer and spring) and EDEN (spring)

**FIGURE 7:**
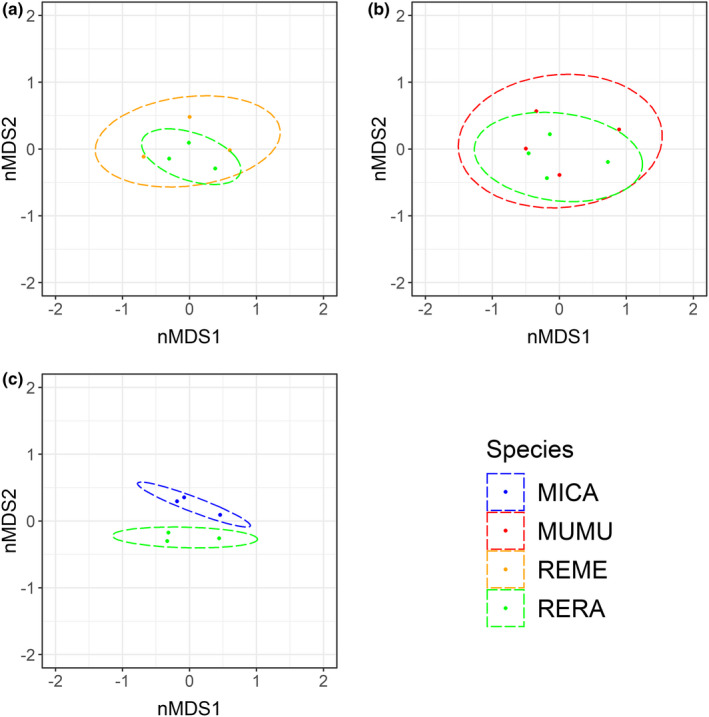
Population‐level dietary overlap as represented in ordination (nMDS) plots of (a) salt marsh harvest mouse (*Reithrodontomys raviventris*; RERA) compared with western harvest mouse (*R. megalotis*; REME), (b) RERA compared with house mouse (*Mus musculus*; MUMU), and (c) RERA compared with California voles (*Microtus californicus*; MICA). Dots represent population‐level diet using frequency of occurrence data. Ellipses show 95% confidence intervals

RERA had significantly lower dietary niche breadth than all three sympatric rodents (Figure 6). Finally, dietary niche overlap in nMDS space was very high with both REME and MUMU, which effectively subsumed RERA dietary niche space (Figure 7a,b). In contrast, ordination highlighted that the diets of RERA and MICA were effectively distinct (Figure 7c).

## 4 DISCUSSION

Salt marsh harvest mouse diet was dominated by *Salicornia* and *Atriplex* year‐round, but also included a wide variety of other native and non‐native plants. Seasonal niche breadth was narrowest in fall when they consumed primarily *Salicornia*, *Atriplex*, and *Grindelia*. RERA diet was less diverse than the diets of sympatric rodents due to less frequent consumption of grasses and upland plants.

### 4.1 Salt marsh harvest mouse diet

Salt marsh harvest mice consumed at least 48 genera of plants in our study. Despite high taxonomic richness in RERA diet, the overwhelming majority was composed of the native *Salicornia* and the non‐native *Atriplex*. *Salicornia* was present in the majority of RERA fecal samples in every sampling unit in our study area, was the most frequently consumed item in six of eight sampling units, and ranked second to *Atriplex* at the other two. *Salicornia* was selected in three of four seasons at Goodyear Slough and across five summer sampling units with varying plant composition. These data support the traditional view that *Salicornia* is a staple in the diet of RERA.

Equally important, however, is that *Atriplex* was nearly as prominent in RERA diet as *Salicornia*. A primary difference between the two plants was the lower availability of *Atriplex*, which led to relatively less consumption overall but high selection coefficients. These data were consistent with cafeteria trials that suggested a strong affinity for *Atriplex* (Smith & Kelt, 2019). In addition, RERA selected several other non‐native plants. The Tidal Marsh Recovery Plan (USFWS, 2010) emphasized conservation concerns associated with the invasion of marshes by non‐native *Lepidium latifolium*. However, RERA consumed *Lepidium* year‐round in proportion to its availability, indicating that low‐to‐moderate availability of this plant did not adversely affect RERA. We did not sample sites where *Lepidium* dominated the vegetative cover, so the impacts of more intense invasions of *Lepidium* remain uninvestigated. Future work to quantify the nutritional value of native and non‐native diet items and their effects on individual survival would provide further clarity on the implications of non‐native plants for RERA population health.

Overall, these data support a hypothesis that *Salicornia* stands including mixtures of plants such as *Atriplex*, *Frankenia*, and *Grindelia* may provide more value to RERA than those with *Salicornia* alone (Fisler, 1965; Shellhammer et al., 1982). In particular, a growing body of work from Suisun Marsh, where brackish water promotes more diverse plant communities, has emphasized the importance of mixed vegetative communities over *Salicornia*‐dominated sites (Botti et al., 1986; Smith & Kelt, 2019; Sustaita et al., 2011). Our data clarified that *Salicornia* is an important element in RERA diet, but that their diets were not strictly specialized.

### 4.2 Seasonal changes in salt marsh harvest mouse diet

Optimal foraging theory suggests that animals will specialize on preferred foods when they are available, and that they will broaden their diets when preferred foods are unavailable (MacArthur & Pianka, 1966; Stephens & Krebs, 1986). In fall, RERA diet narrowed sharply and was overwhelmingly composed of three species (*Salicornia*, *Atriplex*, and *Grindelia*). In spring, however, consumption of these three plants declined and their dietary breadth expanded accordingly. We suspect that RERA foraging patterns may largely be driven by affinities for these three plants. Dietary seasonality, in turn, likely is driven by plant phenology. Fall, when RERA diet narrowed to focus almost exclusively on *Salicornia*, *Atriplex*, and *Grindelia*, is the peak seeding period for these three plants (Hutchings & Russell, 1989; Jepson eFlora Project et al., 2021), and is followed by dormancy or dieback in late winter and early spring, which coincided with reduced consumption by RERA. Whereas annual dieback of *Atriplex* has led some to suggest that this plant has limited value to RERA in winter and spring (Botti et al., 1986; USFWS, 2010), our data suggest substantial consumption of *Atriplex* year‐round despite seasonal dieback. In contrast, some non‐native plants, such as *Phragmites*, were consumed primarily during one season, and were avoided most of the year. It is possible that *Phragmites* seeds do not persist in the environment as long as *Atriplex*, thus limiting their seasonal availability as forage.

Seasonal space use may play an important role in seasonal dietary patterns of RERA. *Grindelia* provides refuge for RERA during high tides (USFWS, 2010). In particular, RERA often seek refuge in emergent *Grindelia* during extreme diurnal high tide events in late fall and early winter, whereas other rodents are more likely to retreat to uplands (Johnston, 1957). We observed higher frequencies of *Grindelia* in RERA diets during fall and winter, which may reflect an increase in habitat use associated with seasonally high tides. Taken together, these observations suggest that *Grindelia* may provide an important combination of high tide refuge, cover from predators, and forage to RERA during extreme diurnal high tides of late fall and early winter.

The diet of RERA broadened in spring, with increased consumption of upland plants that were negligible in the diets in other seasons. This was particularly notable for upland grasses, which is consistent with previous RERA stomach content analyses (Fisler, 1965). RERA remain largely restricted to marsh habitat with the exception of spring forays into terrestrial grasslands (Shellhammer et al., 1982; USFWS, 2010; Zetterquist, 1977). Fisler (1965) speculated that vegetative cover in grasslands was insufficient for RERA outside the spring growing season. Geissel et al. (1988) suggested that RERA retreated to uplands in response to springtime population irruptions of larger bodied voles. Although we cannot discern whether competition or seasonal resource exploitation drove this pattern, our data support the hypothesis that utilization of terrestrial grasslands by RERA is largely limited to spring.

Several seasonal patterns in our data mirrored observations from cafeteria trials (Smith & Kelt, 2019). Seasonal selection indices of *Salicornia* and *Atriplex* in our study were high in fall and low in spring, corresponding with seasonal preference rankings in cafeteria trials. In contrast, our data showed high FO of these plants in summer as well, whereas feeding trials did not. Our data also aligned with feeding trials that suggested increased preference for annual grasses in spring. On the other hand, feeding trials suggested high or moderate preference for *Juncus* in multiple seasons; we found low consumption of *Juncus* both overall and in proportion to availability in the present study. Despite high availability and high FO in MICA diet, we detected *Juncus* in just one of 189 RERA samples at Goodyear Slough. Another major conclusion from feeding trials was a strong preference for *Polypogon*. Our ability to corroborate this result may have been limited by low availability or absence of this plant from most of our study sites. *Polypogon* was rare at Goodyear Slough and relatively common at Crescent Unit, and consumption by RERA occurred in proportion to its availability.

### 4.3 Dietary comparisons to co‐occurring rodents

Diet of the endangered RERA overlapped substantially with that of the widespread REME, driven primarily by high frequencies of *Salicornia* and *Atriplex*. Although the kidney physiology of REME suggests capability to consume *Salicornia*, they were unable to survive in feeding trials after consuming even small amounts of this plant (Coulombe, 1970). Similarly, captive REME starved when presented with only *Salicornia* and *Distichlis* as food sources (Fisler, 1965). Nonetheless, our data revealed that wild REME regularly consumed both of these genera (Figure 5, Appendix A9). REME were the only species to consume *Atriplex* (which grows primarily in diked wetlands) more frequently than *Salicornia* (which occurs frequently in both diked and tidal wetlands). This pattern most likely reflects differential space use, as REME are more abundant on diked wetlands than tidal wetlands, and RERA and MUMU abundances do not differ among wetland types (Smith et al., 2020). REME also consumed grasses (*Distichlis*, *Festuca*, *Hordeum*, and *Phragmites*) and upland plants such as thistles (*Sonchus* and Cynareae) with greater frequency than did RERA.

We also documented considerable dietary overlap between MUMU and RERA, driven by high frequencies of *Salicornia* and *Atriplex*. Similar to REME, MUMU consumed more grasses (*Distichlis*, *Phragmites*, *Hordeum*, and *Festuca*) and upland plants (*Sonchus*, Cynareae) than did RERA. In studies of habitat use in the SFE, MUMU were more closely associated with terrestrial grasses and fragmented habitat assemblages than were RERA (Bias, 1994; Bias & Morrison, 2006). Interestingly, despite a relatively generalist diet, only a single house mouse (of 26) consumed *Grindelia*, which was one of the most frequently consumed plants for the three native rodents (Figure 5, Appendix A9).

Relative to RERA, the most distinct diet was that of MICA, primarily due to reduced use of *Atriplex* and a high frequency of *Juncus*. Although few plant species were consumed by a single rodent species in our study, MICA was the only species to utilize *Juncus* to a great extent. Despite being characterized as grassland specialists, MICA in our study consumed lower frequencies of terrestrial grasses than either REME or MUMU. Instead, MICA diet was dominated by *Salicornia*, *Juncus*, and *Distichlis*, differing from more upland locations in the SFE, where they primarily consume terrestrial grasses (Batzli & Pitelka, 1971). In fact, the diet of MICA in our study more closely resembled that of Amargosa voles (*M. c. scirpensis*), a subspecies endemic to wetlands in the Mohave Desert (Castle et al., 2020), than MICA occupying the uplands adjacent to SFE marshes (Batzli & Pitelka, 1971).

The diet of RERA was more restricted (Figure 5) and significantly less diverse (Figure 6) than that of sympatric species. Preference for *Salicornia* and *Atriplex* was notably greater for RERA, while sympatric species consumed higher proportions of several other species (Figure 5). Notably, many of these latter plants were restricted to uplands, indicating that sympatric rodents are better equipped than RERA to utilize resources in edge habitats. Indeed, we note that REME, MUMU, and MICA generally are considered upland species, thus, our characterizations of their diets is specific to the individuals occurring on the upland/marshland edges and likely not reflective of these species as a whole. Habitat fragmentation and small patch size reduce the probability of RERA occurrence (Bias & Morrison, 2006; Marcot et al., 2020), and occupancy of marsh habitat by REME and MUMU may be dependent upon the degree of habitat fragmentation and penetration of terrestrial grass microhabitats into the marsh (Bias & Morrison, 2006; Fisler, 1965). Our results support these important management issues, adding to a growing literature suggesting that fragmentation of marsh habitat and the associated increase in edge habitat are potential threats to RERA with respect to competition from upland‐adapted rodents.

## 5 CONCLUSIONS

We characterized the diet of RERA and three sympatric rodents in remnant coastal marsh habitat of the SFE. *Salicornia* and *Atriplex* were prominent in RERA diet across sites and seasons. RERA diet narrowed sharply in fall during peak seed production of *Salicornia*, *Atriplex*, and *Grindelia*, which appeared to be favored food items. RERA consumption of terrestrial grass was largely restricted to spring, coinciding with previously documented patterns of seasonal use of upland habitats. RERA diet overlapped substantially with REME and the non‐native MUMU, but not with the native MICA. Our data provide the first comprehensive characterization of RERA diet in the wild. This information fills critical knowledge gaps in the ecology of RERA and can guide habitat and vegetation management decisions to benefit conservation of the species. Moreover, our study lays the groundwork for future investigation of competition affecting this endangered species.

## AUTHOR CONTRIBUTIONS

**Cody M. Aylward:** Conceptualization (lead); data curation (lead); formal analysis (lead); funding acquisition (supporting); methodology (equal); visualization (lead); writing – original draft (lead); writing – review and editing (equal). **Mark Statham:** Conceptualization (equal); data curation (supporting); funding acquisition (supporting); methodology (equal); writing – review and editing (equal). **Laureen Barthman‐Thompson:** Conceptualization (supporting); data curation (supporting); writing – review and editing (equal). **Douglas Kelt:** Conceptualization (equal); funding acquisition (lead); methodology (equal); writing – review and editing (equal). **Benjamin Sacks:** Conceptualization (equal); methodology (equal); writing – review and editing (equal).

## CONFLICT OF INTEREST

The authors declare no conflict of interest.

## Supporting information


Appendix S1

Appendix S2

## Data Availability

Sequence and sample data are published on Dryad (https://doi.org/10.5061/dryad.9w0vt4bjd).
