# Supplementary material for: Dietary characterization of the endangered salt marsh harvest mouse and sympatric rodents using DNA metabarcoding
Source: Ecol Evol. 2022 Jul 17;12(7):e9121. doi: 10.1002/ece3.9121 (PMC9289124; doi:10.1002/ece3.9121)
Supplement: Supplementary file 1 — Appendix S1 [file ECE3-12-e9121-s002.docx]

**Appendix A**

Table A1. Primer sequences used in this study. In complete primer sequences, brackets denote the sequence of the 5’ overhang used for annealing to indexed adapters for Illumina sequencing. Note that these overhangs were complimentary to indexed adapters developed in-house and do not necessarily anneal to commercial indexed adapters. Nucleotide sequences following bracketed sequences represent the nucleotide sequences from referenced literature.

| Amplicon Primer Name | Complete Primer Sequence | Reference |
| --- | --- | --- |
| trnl_g | 5-[TCTTTCCCTACACGACGCTCTTCCGATC]GGGCAATCCTGAGCCAA-3 | Taberlet et al. 2007 |
| trnl_h | 5-[GTGACTGGAGTTCAGACGTGTGCTCTTCCGATC]CCATTGAGTCTCTGCACCTATC-3 | Taberlet et al. 2007 |
| UniPlantF | 5-[TCTTTCCCTACACGACGCTCTTCCGATC]TGTGAATTGCARRATYCMG-3 | Moorhouse-Gann et al. 2018 |
| ITS-p4 | 5-[GTGACTGGAGTTCAGACGTGTGCTCTTCCGATC]CCGCTTAKTGATATGCTTAAA-3 | Cheng et al. 2016 |

Table A2. *in silico* PCR penalty scores of primer pairs used in our study against sequences of select taxa downloaded from Genbank. Scores were calculated using the *evaluate_primer* tool in the R package ‘PrimerMiner’, with default settings. Sequences that produced failing scores (>120) are denoted by an asterisk (*). Failing scores did not appear systemic for any taxon. *Schoenoplectus* was not amplified by either of the two primer pairs despite strong *in silico* scores.

|  | ITS2 | | |  |  | trnL | | |
| --- | --- | --- | --- | --- | --- | --- | --- | --- |
| Template | UniPlantF | ITS-p4 | Score |  | Template | trnl_g | trnl_h | Score |
| *Atriplex* 1 | 0 | 0 | 0 |  | *Atriplex* 1 | 0 | 24.8 | 24.8 |
| *Atriplex* 2 | 0 | 0 | 0 |  | *Atriplex* 2 | 0 | 24.8 | 24.8 |
| *Atriplex* 3 | 0 | 0 | 0 |  | *Atriplex* 3 | 0 | 24.8 | 24.8 |
| *Atriplex* 4 | 0 | 0 | 0 |  | *Bromus* 1 | 0 | 0 | 0 |
| *Atriplex* 5 | 0 | 0 | 0 |  | *Bromus* 2 | 0 | 0 | 0 |
| *Atriplex* 6* | 0 | 174.7 | 174.7 |  | *Bromus* 3 | 0 | 0 | 0 |
| *Atriplex* 7 | 0 | 0 | 0 |  | *Cotula* 1 | 0 | 7.8 | 7.8 |
| *Bolboschoenus* 1 | 0 | 73.8 | 73.8 |  | *Cuscuta* 1 | 0 | 6.2 | 6.2 |
| *Bromus* 1 | 0 | 73.8 | 73.8 |  | *Cuscuta* 2 | 0 | 6.2 | 6.2 |
| *Bromus* 2 | 0 | 73.8 | 73.8 |  | *Cuscuta* 3*** | 0 | 345.8 | 345.8 |
| *Cotula* 1 | 0 | 0 | 0 |  | *Cuscuta* 4 | 0 | 6.2 | 6.2 |
| *Cotula* 1 | 0 | 0 | 0 |  | *Distichlis* 1 | 0 | 0 | 0 |
| *Cuscuta* 1 | 0 | 0 | 0 |  | *Distichlis* 2 | 0 | 0 | 0 |
| *Cuscuta* 2 | 0 | 0 | 0 |  | *Distichlis* 3 | 0 | 0 | 0 |
| *Cuscuta* 3 | 0 | 0 | 0 |  | *Festuca* 1 | 0 | 0 | 0 |
| *Distichlis* 1 | 0 | 0 | 0 |  | *Festuca* 2 | 0 | 0 | 0 |
| *Distichlis* 2 | 0 | 0 | 0 |  | *Grindelia* 1 | 0 | 7.8 | 7.8 |
| *Distichlis* 3 | 0 | 0 | 0 |  | *Juncus* 1 | 0 | 6.2 | 6.2 |
| *Distichlis* 4 | 0 | 0 | 0 |  | *Juncus* 2 | 0 | 6.2 | 6.2 |
| *Festuca* 1 | 0 | 73.8 | 73.8 |  | *Juncus* 3 | 0 | 6.2 | 6.2 |
| *Festuca* 2 | 0 | 0 | 0 |  | *Juncus* 4 | 0 | 6.2 | 6.2 |
| *Festuca* 3 | 0 | 0 | 0 |  | *Juncus* 5 | 0 | 6.2 | 6.2 |
| *Frankenia* 1 | 0 | 0 | 0 |  | *Lepidium* 1 | 0 | 0 | 0 |
| *Grindelia* 1 | 0 | 0 | 0 |  | *Lepidium* 2 | 0 | 0 | 0 |
| *Juncus* 1 | 0 | 94.2 | 94.2 |  | *Phragmites* 1 | 0 | 0 | 0 |
| *Juncus* 2 | 0 | 94.2 | 94.2 |  | *Polypogon* 1 | 0 | 0 | 0 |
| *Juncus* 3 | 0 | 94.2 | 94.2 |  | *Polypogon* 2 | 0 | 0 | 0 |
| *Lepidium* 1 | 0 | 0 | 0 |  | *Salicornia* 1 | 0 | 24.8 | 24.8 |
| *Lepidium* 2 | 0 | 0 | 0 |  | *Salicornia* 2 | 0 | 24.8 | 24.8 |
| *Lepidium* 3 | 0 | 0 | 0 |  | *Salicornia* 3 | 0 | 24.8 | 24.8 |
| *Phragmites* 1 | 0 | 73.8 | 73.8 |  | *Salicornia* 4 | 0 | 24.8 | 24.8 |
| *Polypogon* 1 | 0 | 73.8 | 73.8 |  | *Salicornia* 5 | 0 | 24.8 | 24.8 |
| *Polypogon* 2 | 0 | 73.8 | 73.8 |  | *Schoenoplectus* 1 | 0 | 7.8 | 7.8 |
| *Salicornia* 1 | 0 | 0 | 0 |  | *Schoenoplectus* 2 | 73.8 | 7.8 | 81.6 |
| *Salicornia* 2 | 0 | 105.2 | 105.2 |  | *Schoenoplectus* 3 | 0 | 7.8 | 7.8 |
| *Salicornia* 3 | 0 | 0 | 0 |  | *Triglochin* 1 | 0 | 0 | 0 |
| *Salicornia* 4 | 0 | 0 | 0 |  | *Triglochin* 2 | 0 | 0 | 0 |
| *Salicornia* 5 | 0 | 105.2 | 105.2 |  | *Typha* 1 | 0 | 7.8 | 7.8 |
| *Salicornia* 6 | 0 | 0 | 0 |  | *Typha* 2 | 0 | 7.8 | 7.8 |
| *Salsola* 1*** | 0 | 270.9 | 270.9 |  |  |  |  |  |
| *Salsola* 2 | 0 | 0 | 0 |  |  |  |  |  |
| *Schoenoplectus* 1 | 0 | 73.8 | 73.8 |  |  |  |  |  |
| *Schoenoplectus* 2*** | 0 | 134.8 | 134.8 |  |  |  |  |  |
| *Schoenoplectus* 3 | 0 | 73.8 | 73.8 |  |  |  |  |  |
| *Schoenoplectus* 4 | 0 | 0 | 0 |  |  |  |  |  |
| *Triglochin* 1 | 0 | 0 | 0 |  |  |  |  |  |
| *Triglochin* 2 | 0 | 0 | 0 |  |  |  |  |  |
| *Typha* 1 | 0 | 0 | 0 |  |  |  |  |  |

Table A3. Positive controls used during metabarcoding library preparation and sequencing. Four sequencing lanes were used in this study, one per marker per year. In each lane, we used ten single-species controls, with the expectation of recovering 100% of reads from that species, and ten “50-50” controls with two plant species, with the expectation of recovering 50% reads from each species, assuming no amplification bias. Percentages of target species reads shown are post-bioinformatic filtering. Species included in controls include *Grindelia stricta*, *Salicornia pacifica*, *Polypogon monspeliensis*, *Triglochin maritima*, *Schoenoplectus americanus*, *Juncus balticus*, *Lepidium latifolium*, *Bolboschoenus maritima*, *Achillea millefolium*, *Rosa californica*, *Distichlis spicata*, and *Atriplex prostrata*. Although *in silico* PCR scores suggested both primer sets would amplify *Schoenoplectus*, neither produced any sequences that could be identified as *Schoenoplectus* from the positive control. One *Polypogon* positive control produced sequences assigned to *Bromus* in the ITS2 marker, but these two species did not co-occur in any diet sample, so we believe this misassignment did not affect dietary inference.

| 2018-2019 lanes | % target species reads | |  | 2019-2020 lanes | % target species reads | |
| --- | --- | --- | --- | --- | --- | --- |
| Plant Genus | trnL | ITS2 |  | Plant Genus | trnL | ITS2 |
| Single-species controls |  |  |  |  |  |  |
| *Grindelia* | 100 | 100 |  | *Salicornia* | 100 | 100 |
| *Salicornia* | 100 | 100 |  | *Atriplex* | 100 | 100 |
| *Polypogon* | 100 | 100 |  | *Distichlis* | 100 | 100 |
| *Triglochin* | 100 | 100 |  | *Grindelia* | 100 | 100 |
| *Schoenoplectus* | 0 | 0 |  | *Schoenoplectus* | 0 | 0 |
| *Juncus* | 100 | 0 |  | *Lepidium* | 100 | 100 |
| *Lepidium* | 100 | 100 |  | *Bolboschoenus* | 100 | 0 |
| *Bolboschoenus* | 100 | 100 |  | *Juncus* | 100 | 0 |
| *Achillea* | 100 | 100 |  | *Polypogon* | 100 | 64 |
| *Rosa* | 100 | 100 |  | *Frankenia* | 100 | 100 |
| 50-50 controls |  |  |  |  |  |  |
| *Grindelia/Salicornia* | 53/47 | 51/49 |  | *Salicornia/Atriplex* | 60/40 | 50/50 |
| *Polypogon/Triglochin* | 88/12 | 23/77 |  | *Distichlis/Grindelia* | 49/51 | 30/70 |
| *Juncus/Schoenoplectus* | 100/0 | 0/0 |  | *Schoenoplectus/Lepidium* | 0/100 | 0/100 |
| *Bolboschoenus/Lepidium* | 2/98 | 17/83 |  | *Bolboschoenus/Juncus* | 2/98 | 0/0 |
| *Achillea/Rosa* | 17/83 | 64/36 |  | *Polypogon/Frankenia* | 67/33 | 3/97 |
| *Grindelia/Polypogon* | 60/40 | 94/6 |  | *Salicornia/Lepidium* | 32/68 | 41/59 |
| *Salicornia/Triglochin* | 93/7 | 50/50 |  | *Atriplex/Bolboschoenus* | 93/7 | 98/2 |
| *Schoenoplectus/Lepidium* | 0/100 | 0/100 |  | *Distichlis/Juncus* | 35/65 | 100/0 |
| *Juncus/Achillea* | 72/28 | 0/100 |  | *Grindelia/Polypogon* | 59/41 | 95/5 |
| *Bolboschoenus/Rosa* | 3/97 | 7/93 |  | *Schoenoplectus/Frankenia* | 0/100 | 0/100 |

Table A4. Correlation coefficient (*r*) of Frequency of Occurrence (FO) data and Relative Read Abundance (RRA) data within each marker data set for salt marsh harvest mice (*Reithrodontomys raviventris*; RERA), western harvest mice (*R. megalotis*; REME), house mice (*Mus musculus*; MUMU) and California voles (*Microtus californicus*; MICA). Correlation strength scaled with sample size and reached high levels even in the rodent species with the smallest sample size (*n* = 20) in our study.

|  |  | *r* of FO and RRA | |
| --- | --- | --- | --- |
| Species | n | trnL | ITS2 |
| RERA | 245 | 0.978 | 0.991 |
| REME | 30 | 0.944 | 0.986 |
| MUMU | 26 | 0.934 | 0.960 |
| MICA | 20 | 0.888 | 0.937 |

Table A5. Frequency of Occurrence (FO) of diet items in salt marsh harvest mouse (*Reithrodontomys raviventris*) diet (*n* = 245) pooled across all sites and seasons. * = non-native taxa.

| Taxon | FO | Taxon | FO |
| --- | --- | --- | --- |
| *Salicornia* | 0.743 | *Baccharis* | 0.012 |
| *Atriplex** | 0.563 | *Cordylanthus* | 0.012 |
| *Distichlis* | 0.224 | *Foeniculum** | 0.012 |
| *Grindelia* | 0.200 | *Lactuca** | 0.012 |
| *Rumex** | 0.139 | *Salsola** | 0.012 |
| *Lepidium** | 0.127 | *Apium** | 0.008 |
| *Phragmites** | 0.122 | *Carduus** | 0.008 |
| Polygonaceae | 0.073 | *Chenopodium** | 0.008 |
| *Cuscuta* | 0.065 | *Conium** | 0.008 |
| *Cotula** | 0.057 | *Elymus** | 0.008 |
| *Frankenia* | 0.057 | *Glaux* | 0.008 |
| *Hordeum** | 0.045 | *Juncus* | 0.008 |
| *Baccharis/Euthamia* | 0.041 | *Potentilla* | 0.008 |
| Cynareae* | 0.041 | *Sambucus* | 0.008 |
| *Sonchus** | 0.041 | *Bolboschoenus* | 0.004 |
| *Lotus** | 0.033 | *Cressa* | 0.004 |
| *Parapholis* | 0.033 | *Euthamia* | 0.004 |
| Convolvulaceae | 0.029 | *Geranium** | 0.004 |
| *Festuca** | 0.029 | *Hainardia** | 0.004 |
| *Jaumea* | 0.029 | *Lathyrus* | 0.004 |
| *Spergularia* | 0.029 | *Matricaria* | 0.004 |
| *Achillea* | 0.024 | *Mesembryanthemum* | 0.004 |
| *Typha* | 0.024 | *Polygonum* | 0.004 |
| *Echinochloa** | 0.020 | *Raphanus** | 0.004 |
| *Polypogon** | 0.020 | Rosaceae | 0.004 |
| *Triglochin* | 0.020 | *Trifolium* | 0.004 |
| *Solanum* | 0.016 |  |  |

Table A6. Frequency of Occurrence (FO) of plant forms in salt marsh harvest mouse (*Reithrodontomys raviventris*) diet pooled across all sites and seasons (*n* = 245). Plant forms and habitats were determined from CalFlora.org.

|  | Plant Form | | | | | |
| --- | --- | --- | --- | --- | --- | --- |
| Season | Forb/Subshrub (Wetland) | Grass (Wetland) | Forb/Subshrub (Upland) | Grass (Upland) | Vine | Shrub |
| Summer | 0.933 | 0.689 | 0.200 | 0.067 | 0.089 | 0.000 |
| Fall | 1.000 | 0.096 | 0.038 | 0.000 | 0.077 | 0.000 |
| Winter | 0.958 | 0.521 | 0.063 | 0.000 | 0.271 | 0.000 |
| Spring | 0.932 | 0.341 | 0.295 | 0.318 | 0.045 | 0.045 |
| Overall | 0.958 | 0.402 | 0.143 | 0.090 | 0.122 | 0.011 |

Table A7. Seasonal Frequency of Occurrence (FO) of plant taxa in salt marsh harvest mouse (*Reithrodontomys raviventris*) diet in four seasons at Goodyear Slough.

| Diet Taxon | Summer | Fall | Winter | Spring | Overall |
| --- | --- | --- | --- | --- | --- |
| *Salicornia* | 0.778 | 0.846 | 0.771 | 0.500 | 0.730 |
| *Atriplex* | 0.556 | 0.827 | 0.646 | 0.545 | 0.651 |
| *Distichlis* | 0.311 | 0.038 | 0.375 | 0.341 | 0.259 |
| *Grindelia* | 0.156 | 0.558 | 0.250 | 0.023 | 0.259 |
| *Rumex* | 0.244 | 0.000 | 0.229 | 0.227 | 0.169 |
| *Phragmites* | 0.533 | 0.019 | 0.104 | 0.000 | 0.159 |
| *Lepidium* | 0.178 | 0.077 | 0.125 | 0.182 | 0.138 |
| *Cuscuta* | 0.044 | 0.058 | 0.229 | 0.000 | 0.085 |
| Polygonaceae | 0.244 | 0.038 | 0.063 | 0.000 | 0.085 |
| *Baccharis/Euthamia* | 0.044 | 0.019 | 0.104 | 0.045 | 0.053 |
| *Hordeum* | 0.044 | 0.000 | 0.000 | 0.159 | 0.048 |
| Cynareae | 0.044 | 0.000 | 0.000 | 0.136 | 0.042 |
| Convolvulaceae | 0.044 | 0.019 | 0.042 | 0.045 | 0.037 |
| *Cotula* | 0.089 | 0.000 | 0.000 | 0.068 | 0.037 |
| *Sonchus* | 0.089 | 0.000 | 0.000 | 0.068 | 0.037 |
| *Achillea* | 0.089 | 0.019 | 0.021 | 0.000 | 0.032 |
| *Jaumea* | 0.000 | 0.038 | 0.063 | 0.023 | 0.032 |
| *Typha* | 0.022 | 0.038 | 0.021 | 0.045 | 0.032 |
| *Festuca* | 0.000 | 0.000 | 0.000 | 0.114 | 0.026 |
| *Echinochloa* | 0.000 | 0.019 | 0.063 | 0.000 | 0.021 |
| *Baccharis* | 0.022 | 0.038 | 0.000 | 0.000 | 0.016 |
| *Foeniculum* | 0.067 | 0.000 | 0.000 | 0.000 | 0.016 |
| *Lactuca* | 0.022 | 0.000 | 0.042 | 0.000 | 0.016 |
| *Parapholis* | 0.000 | 0.000 | 0.000 | 0.068 | 0.016 |
| *Solanum* | 0.022 | 0.019 | 0.000 | 0.023 | 0.016 |
| *Carduus* | 0.000 | 0.000 | 0.000 | 0.045 | 0.011 |
| *Conium* | 0.000 | 0.000 | 0.000 | 0.045 | 0.011 |
| *Juncus* | 0.000 | 0.000 | 0.021 | 0.023 | 0.011 |
| *Lotus* | 0.000 | 0.000 | 0.000 | 0.045 | 0.011 |
| *Potentilla* | 0.022 | 0.000 | 0.000 | 0.023 | 0.011 |
| *Salsola* | 0.000 | 0.000 | 0.021 | 0.023 | 0.011 |
| *Sambucus* | 0.000 | 0.000 | 0.000 | 0.045 | 0.011 |
| *Triglochin* | 0.000 | 0.000 | 0.000 | 0.045 | 0.011 |
| *Apium* | 0.000 | 0.000 | 0.021 | 0.000 | 0.005 |
| *Bolboschoenus* | 0.000 | 0.019 | 0.000 | 0.000 | 0.005 |
| *Chenopodium* | 0.000 | 0.000 | 0.000 | 0.023 | 0.005 |
| *Elymus* | 0.000 | 0.000 | 0.000 | 0.023 | 0.005 |
| *Euthamia* | 0.000 | 0.019 | 0.000 | 0.000 | 0.005 |
| *Hainardia* | 0.000 | 0.000 | 0.000 | 0.023 | 0.005 |
| *Lathyrus* | 0.000 | 0.000 | 0.000 | 0.023 | 0.005 |
| *Matricaria* | 0.000 | 0.000 | 0.021 | 0.000 | 0.005 |
| *Polypogon* | 0.022 | 0.000 | 0.000 | 0.000 | 0.005 |
| *Raphanus* | 0.000 | 0.000 | 0.000 | 0.023 | 0.005 |
| Rosaceae | 0.000 | 0.019 | 0.000 | 0.000 | 0.005 |
| *Trifolium* | 0.000 | 0.019 | 0.000 | 0.000 | 0.005 |

Table A8. Spatial variation in Frequency of Occurrence (FO) of taxa in salt marsh harvest mouse (*Reithrodontomys raviventris*) diet at five sampling units surveyed in late spring or summer: Crescent Unit (CRES), Eden Landing (EDEN), Goodyear Slough (GYS; summer), Hill Slough 1&2 (HS12), and Hill Slough 9 (HS9).

|  | Site | | | | | Mean |
| --- | --- | --- | --- | --- | --- | --- |
| Diet Taxon | CRES | EDEN | GYS (Su) | HS12 | HS9 |  |
| *Salicornia* | 0.923 | 0.952 | 0.778 | 0.625 | 0.500 | 0.756 |
| *Atriplex* | 0.308 | 0.048 | 0.556 | 0.875 | 0.214 | 0.400 |
| *Frankenia* | 0.000 | 0.429 | 0.000 | 0.500 | 0.071 | 0.200 |
| *Distichlis* | 0.000 | 0.000 | 0.311 | 0.250 | 0.286 | 0.169 |
| *Lepidium* | 0.000 | 0.000 | 0.178 | 0.500 | 0.071 | 0.150 |
| *Cotula* | 0.462 | 0.000 | 0.089 | 0.125 | 0.000 | 0.135 |
| *Phragmites* | 0.000 | 0.000 | 0.533 | 0.000 | 0.000 | 0.107 |
| Polygonaceae | 0.000 | 0.000 | 0.244 | 0.125 | 0.071 | 0.088 |
| *Lotus* | 0.000 | 0.000 | 0.000 | 0.000 | 0.429 | 0.086 |
| *Rumex* | 0.154 | 0.000 | 0.244 | 0.000 | 0.000 | 0.080 |
| *Sonchus* | 0.077 | 0.048 | 0.089 | 0.125 | 0.000 | 0.068 |
| *Spergularia* | 0.000 | 0.333 | 0.000 | 0.000 | 0.000 | 0.067 |
| *Polypogon* | 0.308 | 0.000 | 0.022 | 0.000 | 0.000 | 0.066 |
| *Parapholis* | 0.000 | 0.190 | 0.000 | 0.000 | 0.071 | 0.052 |
| *Triglochin* | 0.000 | 0.000 | 0.000 | 0.000 | 0.214 | 0.043 |
| *Cordylanthus* | 0.000 | 0.000 | 0.000 | 0.000 | 0.214 | 0.043 |
| *Hordeum* | 0.154 | 0.000 | 0.044 | 0.000 | 0.000 | 0.040 |
| *Grindelia* | 0.000 | 0.000 | 0.156 | 0.000 | 0.000 | 0.031 |
| *Festuca* | 0.154 | 0.000 | 0.000 | 0.000 | 0.000 | 0.031 |
| *Solanum* | 0.000 | 0.000 | 0.022 | 0.125 | 0.000 | 0.029 |
| *Glaux* | 0.000 | 0.000 | 0.000 | 0.000 | 0.143 | 0.029 |
| Cynareae | 0.000 | 0.095 | 0.044 | 0.000 | 0.000 | 0.028 |
| *Chenopodium* | 0.000 | 0.000 | 0.000 | 0.125 | 0.000 | 0.025 |
| *Salsola* | 0.000 | 0.000 | 0.000 | 0.125 | 0.000 | 0.025 |
| *Achillea* | 0.000 | 0.000 | 0.089 | 0.000 | 0.000 | 0.018 |
| *Elymus* | 0.077 | 0.000 | 0.000 | 0.000 | 0.000 | 0.015 |
| *Jaumea* | 0.000 | 0.000 | 0.000 | 0.000 | 0.071 | 0.014 |
| *Apium* | 0.000 | 0.000 | 0.000 | 0.000 | 0.071 | 0.014 |
| *Polygonum* | 0.000 | 0.000 | 0.000 | 0.000 | 0.071 | 0.014 |
| *Foeniculum* | 0.000 | 0.000 | 0.067 | 0.000 | 0.000 | 0.013 |
| *Cressa* | 0.000 | 0.048 | 0.000 | 0.000 | 0.000 | 0.010 |
| *Echinochloa* | 0.000 | 0.048 | 0.000 | 0.000 | 0.000 | 0.010 |
| *Geranium* | 0.000 | 0.048 | 0.000 | 0.000 | 0.000 | 0.010 |
| *Mesembryanthemum* | 0.000 | 0.048 | 0.000 | 0.000 | 0.000 | 0.010 |
| *Baccharis/Euthamia* | 0.000 | 0.000 | 0.044 | 0.000 | 0.000 | 0.009 |
| Convolvulaceae | 0.000 | 0.000 | 0.044 | 0.000 | 0.000 | 0.009 |
| *Cuscuta* | 0.000 | 0.000 | 0.044 | 0.000 | 0.000 | 0.009 |
| *Typha* | 0.000 | 0.000 | 0.022 | 0.000 | 0.000 | 0.004 |
| *Potentilla* | 0.000 | 0.000 | 0.022 | 0.000 | 0.000 | 0.004 |
| *Baccharis* | 0.000 | 0.000 | 0.022 | 0.000 | 0.000 | 0.004 |
| *Lactuca* | 0.000 | 0.000 | 0.022 | 0.000 | 0.000 | 0.004 |

Table A9. Frequency of diet items for salt marsh harvest mice (*Reithrodontomys raviventris*; RERA), western harvest mice (*R. megalotis*; REME), house mice (*Mus musculus*; MUMU), and California voles (*Microtus californicus*; MICA). Data were pooled across all sites and seasons. Sample sizes were significantly weighted toward Goodyear Slough (*n* = 245 out of 327), thus pooled frequencies are biased against some taxa that were absent from Goodyear Slough but prominent elsewhere (e.g., *Frankenia*).

| Diet Item | RERA | REME | MUMU | MICA |
| --- | --- | --- | --- | --- |
| *Salicornia* | 0.743 | 0.567 | 0.692 | 0.850 |
| *Atriplex* | 0.563 | 0.733 | 0.500 | 0.150 |
| *Distichlis* | 0.224 | 0.367 | 0.346 | 0.300 |
| *Grindelia* | 0.200 | 0.200 | 0.038 | 0.300 |
| *Phragmites* | 0.122 | 0.333 | 0.231 | 0.050 |
| *Juncus* | 0.008 | 0 | 0 | 0.500 |
| *Hordeum* | 0.045 | 0.133 | 0.231 | 0.050 |
| *Baccharis/Euthamia* | 0.041 | 0.100 | 0 | 0.300 |
| *Frankenia* | 0.057 | 0 | 0.115 | 0.250 |
| Cynareae | 0.041 | 0.100 | 0.077 | 0.200 |
| *Sonchus* | 0.041 | 0.100 | 0.115 | 0.150 |
| *Rumex* | 0.139 | 0.033 | 0.077 | 0.100 |
| Convolvulaceae | 0.029 | 0.033 | 0.077 | 0.200 |
| *Festuca* | 0.029 | 0.100 | 0.192 | 0 |
| *Lepidium* | 0.127 | 0.067 | 0.077 | 0.050 |
| Polygonaceae | 0.073 | 0.167 | 0.038 | 0 |
| *Cotula* | 0.057 | 0 | 0.115 | 0.100 |
| *Cuscuta* | 0.065 | 0.100 | 0.038 | 0.050 |
| *Potentilla* | 0.008 | 0 | 0 | 0.200 |
| *Achillea* | 0.024 | 0.033 | 0 | 0.150 |
| *Spergularia* | 0.029 | 0 | 0.077 | 0.100 |
| *Jaumea* | 0.029 | 0.067 | 0 | 0.100 |
| *Euthamia* | 0.004 | 0 | 0 | 0.150 |
| *Asparagus* | 0 | 0 | 0 | 0.150 |
| *Sinapis* | 0 | 0 | 0.038 | 0.100 |
| *Calystegia* | 0 | 0.033 | 0 | 0.100 |
| *Bromus* | 0 | 0 | 0.077 | 0.050 |
| *Raphanus* | 0.004 | 0.067 | 0.038 | 0 |
| *Sambucus* | 0.008 | 0 | 0 | 0.100 |
| *Typha* | 0.024 | 0 | 0.077 | 0 |
| *Baccharis* | 0.012 | 0.033 | 0 | 0.050 |
| *Apium* | 0.008 | 0.033 | 0 | 0.050 |
| *Brassica* | 0 | 0 | 0.038 | 0.050 |
| *Cressa* | 0.004 | 0.033 | 0 | 0.050 |
| *Foeniculum* | 0.012 | 0.067 | 0 | 0 |
| *Parapholis* | 0.033 | 0 | 0.038 | 0 |
| *Polygonum* | 0.004 | 0.067 | 0 | 0 |
| *Lactuca* | 0.012 | 0 | 0 | 0.050 |
| *Echinochloa* | 0.020 | 0 | 0.038 | 0 |
| *Polypogon* | 0.020 | 0 | 0.038 | 0 |
| *Salsola* | 0.012 | 0 | 0.038 | 0 |
| *Solanum* | 0.016 | 0.033 | 0 | 0 |
| *Carduus* | 0.008 | 0 | 0.038 | 0 |
| *Chenopodium* | 0.008 | 0 | 0.038 | 0 |
| *Elymus* | 0.008 | 0 | 0.038 | 0 |
| *Bolboschoenus* | 0.004 | 0 | 0.038 | 0 |
| *Mesembryanthemum* | 0.004 | 0 | 0.038 | 0 |
| *Glaux* | 0.008 | 0.033 | 0 | 0 |
| *Lotus* | 0.033 | 0 | 0 | 0 |
| *Triglochin* | 0.020 | 0 | 0 | 0 |
| *Cordylanthus* | 0.012 | 0 | 0 | 0 |
| *Conium* | 0.008 | 0 | 0 | 0 |
| *Geranium* | 0.004 | 0 | 0 | 0 |
| *Hainardia* | 0.004 | 0 | 0 | 0 |
| *Lathyrus* | 0.004 | 0 | 0 | 0 |
| *Matricaria* | 0.004 | 0 | 0 | 0 |
| Rosaceae | 0.004 | 0 | 0 | 0 |
| *Trifolium* | 0.004 | 0 | 0 | 0 |
