# Supplementary material for: Dietary characterization of the endangered salt marsh harvest mouse and sympatric rodents using DNA metabarcoding
Source: Ecol Evol. 2022 Jul 17;12(7):e9121. doi: 10.1002/ece3.9121 (PMC9289124; doi:10.1002/ece3.9121)
Supplement: Supplementary file 2 — Appendix S2 [file ECE3-12-e9121-s001.docx]

**Appendix B: Performance of Metabarcoding Markers**

We used two loci to identify plant items in the diet of salt marsh harvest mice (*Reithrodontomys raviventris*; RERA) and co-occurring rodents. Although dietary metabarcoding studies frequently are performed with a single marker (e.g., trnL: Kartzinel et al. 2015; or ITS2: Iwanowicz et al. 2016), there are clear advantages to using multiple markers. As one example, use of multiple markers targeting a single taxonomic group – plants, in this case – greatly improved the ability to detect the complete taxonomic breadth of plants within the diet of Idaho ground squirrel (*Urocitellus brunneus*; Goldberg et al. 2020).

We assessed the performance of multiple markers targeting the same taxonomic group of diet items using a four-step process. First, we tallied the number of unique amplicon sequence variants (ASVs) detected by each marker (after bioinformatic filtering; thus, “detection” required an ASV to comprise > 0.01% of the reads in a sequencing lane). Second, we calculated the number and proportion of ASVs that were successfully assigned to a plant taxon (as opposed to unassigned reads that we suppose were fungal or bacterial). Third, we determined the number of unique taxa identified by each marker. Finally, we counted the number of samples that were filtered out from each marker’s data set during bioinformatic processing.

We detected 1,226 ASVs in the ITS2 data set, of which 427 (35%) were assigned to a plant taxon (Table A1). We detected 481 ASVs in the trnL data set, of which 211 (44%) were assigned to a plant taxon. Data filtering led to the removal of more ITS2 samples (*n* = 72) than trnL samples (*n* = 9). The majority of discarded samples had too few sequence reads assigned to plant taxa (60% of discards in ITS2; 89% in trnL). We examined the trnL data of the samples that were discarded from the ITS2 dataset to determine whether the ITS2 failures may have been due to taxonomy (e.g., they only contained taxa amplified by our trnL primers). The trnL data of these samples regularly contained plant taxa that were commonly detected in successful ITS2 samples, suggesting that taxonomy was not responsible for ITS2 failures. Overall, only six of 327 (1.7%) samples failed to yield dietary information from both markers. Overall, we detected 62 genera; 13 by ITS2 alone, 12 by trnL alone, and 37 by both markers (Table A2). FO and RRA were strongly correlated within each single-marker data set but were consistently slightly higher for ITS2 (Table S4).

Our study echoed the findings of Goldberg et al. (2020) that multiple markers provide complementary (i.e., not redundant) identification of dietary items, thereby improving the taxonomic breadth of detection in dietary metabarcoding. Furthermore, we found that a substantial and methodical system of positive and negative controls helped to understand the limitations of each marker and design marker-specific bioinformatic filters.

In our study, trnL provided true dietary information for nearly all our samples while approximately 20% of ITS2 samples did not pass bioinformatic filtering procedures. One key advantage that may have contributed to the relative success of trnL in our study is its smaller fragment size (23-92 bp; versus 298-333 bp ITS2), which increased the chances of amplification in severely degraded DNA, such as DNA that has been digested. Furthermore, trnL had a higher proportion of ASVs assigned to plant taxa. Degenerate ITS2 primers, which were selected to increase sensitivity to a broader range of plants, may have been disadvantageous due to the swamping of diet DNA by that of non-target taxa. Nonetheless, our results aligned with previous studies suggesting ITS2 provides finer taxonomic resolution and fewer ambiguous identifications at the genus level (CPBOL Group 2011). Fine taxonomic resolution was important in our study with respect to grasses, which were common in the trapping bait. Several ASVs in the trnL dataset could only be identified as grasses (Poaceae) and were therefore discarded as potentially introduced to RERA diet through bait used in the trapping process, whereas ITS2 had no ambiguous ASVs at the genus level and therefore easily distinguished grass taxa present in bait from grass taxa only present in the wild. For dietary studies that require the use of bait, the fine taxonomic resolution of ITS2 may be a significant advantage to distinguish such taxa from closely related natural components of the target species’ diet. Additionally, although we did not use RRA data due to our multi-marker approach, ITS2 had slightly higher correspondence between RRA and FO for all four of our species’ diets.

Positive and negative controls provided important guidance in determining appropriate bioinformatic filters in our study. Bioinformatic decisions can alter the outcome of metabarcoding studies and are often determined ambiguously. Recent work has highlighted the importance of positive controls to objectively guide computational decisions in metabarcoding (O’Rourke et al. 2020). We used data from our controls to understand the sensitivity of our markers to particular taxa, estimate error rates, identify potential primer biases, and set a threshold number of reads for a sample to be retained/discarded. One interesting finding from our controls was that several taxa that received passing scores in *in silico* PCR tests were unable to be amplified by one or both of our primers. Our results underscore the importance of positive controls to evaluate primer sensitivity and sources of bias in dietary metabarcoding data.

**Appendix B References**

CPBOL Group (2011) Comparative analysis of a large dataset indicates that internal transcribed spacer (ITS) should be incorporated into the core barcode for seed plants. PNAS 108: 19641-19646.

Goldberg AR, Conway CJ, Tank DC, Andrews KR, Gour DS, Waits LP (2020) Diet of a rare herbivore based on DNA metabarcoding of feces: Selection, seasonality, and survival. Ecology and Evolution 10: 7627-7643.

Iwanowicz DD, Vandergast AG, Cornman RS, Adams CR, Kohn JR, Fisher RN, Brehme CS (2016) Metabarcoding of fecal samples to determine herbivore diets: a case study of the endangered Pacific pocket mouse. PLoS ONE 11: e0165366.

Kartzinel TR, Chen PA, Coverdale TC, Erickson DL, Kress WJ, Kuzmina ML, Rubenstein DI, Wang W, Pringle RM (2015) DNA metabarcoding illuminates dietary niche partitioning by African large hervibores. PNAS 112: 8019-8024.

O’Rourke DR, Bokulich NA, Jusino MA, MacManes MD, Foster JT (2020) A total crapshoot? Evaluating bioinformatic decisions in animal diet metabarcoding analyses. Ecology and Evolution 10: 9721-9739.

**Table B1.** Numbers of ASVs, taxa, and samples filtered out from each marker data set. Numbers of ASVs and numbers of plant ASVs are presented for each sequencing lane separately.

|  | trnL | ITS2 |
| --- | --- | --- |
| Number of Reads | 11.5M (2018-19) / 10.7M (2019-20) | 10.4M (2018-19) / 8.6M (2019-20) |
| Number of Plant Reads | 10.5M (91.1%) / 10.6M (99.1%) | 9.2M (89.6%) / 8.2M (95.1%) |
| Number of ASVs | 202 (2018-19) / 179 (2019-20) | 832 (2018-19) / 394 (2019-20) |
| Number of Plant ASVs | 94 (47%) / 117 (65%) | 237 (28%) / 190 (48%) |
| Taxa Detected | 54 | 50 |
| Genera Detected | 49 | 50 |
| Samples Filtered Out | 9 (3%) | 72 (22%) |

**Table B2.** Genera detected by trnL and ITS2 in diets of rodents in our study.

| Genus | trnL | ITS2 |
| --- | --- | --- |
| *Achillea* | X | X |
| *Apium* | X | X |
| *Asparagus* | X | - |
| *Atriplex* | X | X |
| *Baccharis* | - | X |
| *Bolboschoenus* | X | X |
| *Brassica* | X | X |
| *Bromus* | X | X |
| *Calystegia* | - | X |
| *Carduus* | - | X |
| *Chenopodium* | X | - |
| *Conium* | - | X |
| *Cordylanthus* | X | - |
| *Cotula* | X | X |
| *Cressa* | X | - |
| *Cuscuta* | X | X |
| *Distichlis* | X | X |
| *Echinochloa* | X | X |
| *Elymus* | X | X |
| *Epilobium* | X | X |
| *Erodium* | X | - |
| *Euthamia* | - | X |
| *Festuca* | X | X |
| *Foeniculum* | X | X |
| *Frankenia* | X | X |
| *Geranium* | X | X |
| *Glaux* | X | X |
| *Grindelia* | X | X |
| *Hainardia* | - | X |
| *Hordeum* | X | X |
| *Jaumea* | X | X |
| *Juncus* | X | - |
| *Lactuca* | X | X |
| *Lathyrus* | X | X |
| *Lepidium* | X | X |
| *Lotus* | X | X |
| *Lythrum* | X | X |
| *Matricaria* | - | X |
| *Melilotus* | X | - |
| *Mesembryanthemum* | X | - |
| *Parapholis* | X | X |
| *Persicaria* | - | X |
| *Phragmites* | X | X |
| *Polygonum* | X | - |
| *Polypogon* | X | X |
| *Potentilla* | X | X |
| *Raphanus* | X | X |
| *Rosa* | - | X |
| *Rubus* | - | X |
| *Rumex* | X | - |
| *Salicornia* | X | X |
| *Salsola* | X | X |
| *Sambucus* | - | X |
| *Sinapis* | X | X |
| *Solanum* | X | X |
| *Sonchus* | X | X |
| *Sorghum* | X | - |
| *Spergularia* | X | X |
| *Symphyotrichum* | - | X |
| *Trifolium* | X | X |
| *Triglochin* | - | X |
| *Typha* | X | - |
| Family/Multi-Genus |  |  |
| *Baccharis/Euthamia* | X | - |
| Convolvulaceae | X | - |
| Cynareae | X | - |
| Polygonaceae | X | - |
| Rosaceae | X | - |
